# Alpha-helical regions of the protein molecule as organic nanotubes

**DOI:** 10.1186/1556-276X-9-200

**Published:** 2014-05-01

**Authors:** Anatol D Suprun, Liudmyla V Shmeleva

**Affiliations:** 1 Taras Shevchenko National University of Kyiv, Volodymyrska Street, 64/13, Kyiv 01601, Ukraine

**Keywords:** Alpha-helix, Protein, Nanotube

## Abstract

An α-helical region of protein molecule was considered in a model of nanotube. The molecule is in conditions of quantum excitations. Such model corresponds to a one-dimensional molecular nanocrystal with three molecules in an elementary cell at the presence of excitation. For the analysis of different types of conformational response of the α-helical area of the protein molecule on excitation, the nonlinear response of this area to the intramolecular quantum excitation caused by hydrolysis of adenosine triphosphate (ATP) is taken into account. It has been established that in the simplest case, three types of excitation are realized. As estimates show, each of them ‘serves’ different kinds of protein. The symmetrical type of excitation, most likely, is realized in the reduction of traversal-striped skeletal muscles. It has the highest excitation energy. This well protects from casual actions. Antisymmetric excitations have intermediate energy (between symmetrical and asymmetrical). They, most likely, are realized in membranous and nucleic proteins. It is shown that the conformational response of the α-helical region of the protein is (in angstroms) a quantity of order *N*_*c*_/5, where *N*_*c*_ is the number of spiral turns. For the number of turns typical in this case: *N*_*c*_ ~ 10, displacement compounds are a quantity of order 2 Å. It qualitatively corresponds to observable values. Asymmetrical excitations have the lowest energy. Therefore, most likely, they are realized in enzymatic proteins. It was shown that at this type of excitation, the bending of the α-helix is formally directed to the opposite side with respect to the antisymmetric excitations. Also, it has a greater value than the antisymmetric case for *N*_*c*_ ≤ 14 and smaller for *N*_*c*_ > 14.

**PACS:**

92C05

**MCS:**

36.20.Ey

## Background

### Hydrolysis of ATP and amide I excitation

A protein molecule has a rather unique structure not only in the chemical-biological point of view but also as an interesting physical and mathematical object. If we consider it as a physical object, then such object may be referred to as a nanostructure without any doubt. Thus, the alpha-helical region of a protein molecule simultaneously may be considered both as a nanotube and as a nanowire: this depends on the considered level of structure.

Here, the alpha-helix is considered at the level of secondary structure where it is a nanotube. It is in the conditions of quantum excitation which is stimulated by reaction of hydrolysis of adenosine triphosphate (ATP). As a result of this reaction, energy in the form of quanta of infrared range is released. It is considered that they are absorbed by a group of energy states known in an alpha-helix as amide I, etc. It is considered also that these absorbing states have an internally molecular oscillating nature. The results obtained here allow giving a definite answer to this question, because in the infrared range, absorption can also have the nature of electronic transitions between states with the main quantum number equal to 2.

The alpha-helix is interesting as a mathematical object too. Due to the high sensitivity of its ‘crystalline lattice’ in relation to excitation, we are coming to a necessity to solve a nonlinear system of the so-called eigen type, i.e., actually, we are coming to a necessity to search for the eigenvalues and eigenvectors of a nonlinear system of algebraic equations. Such a problem, as it is known to us, is a scantily explored mathematical problem.

**Figure 1:**
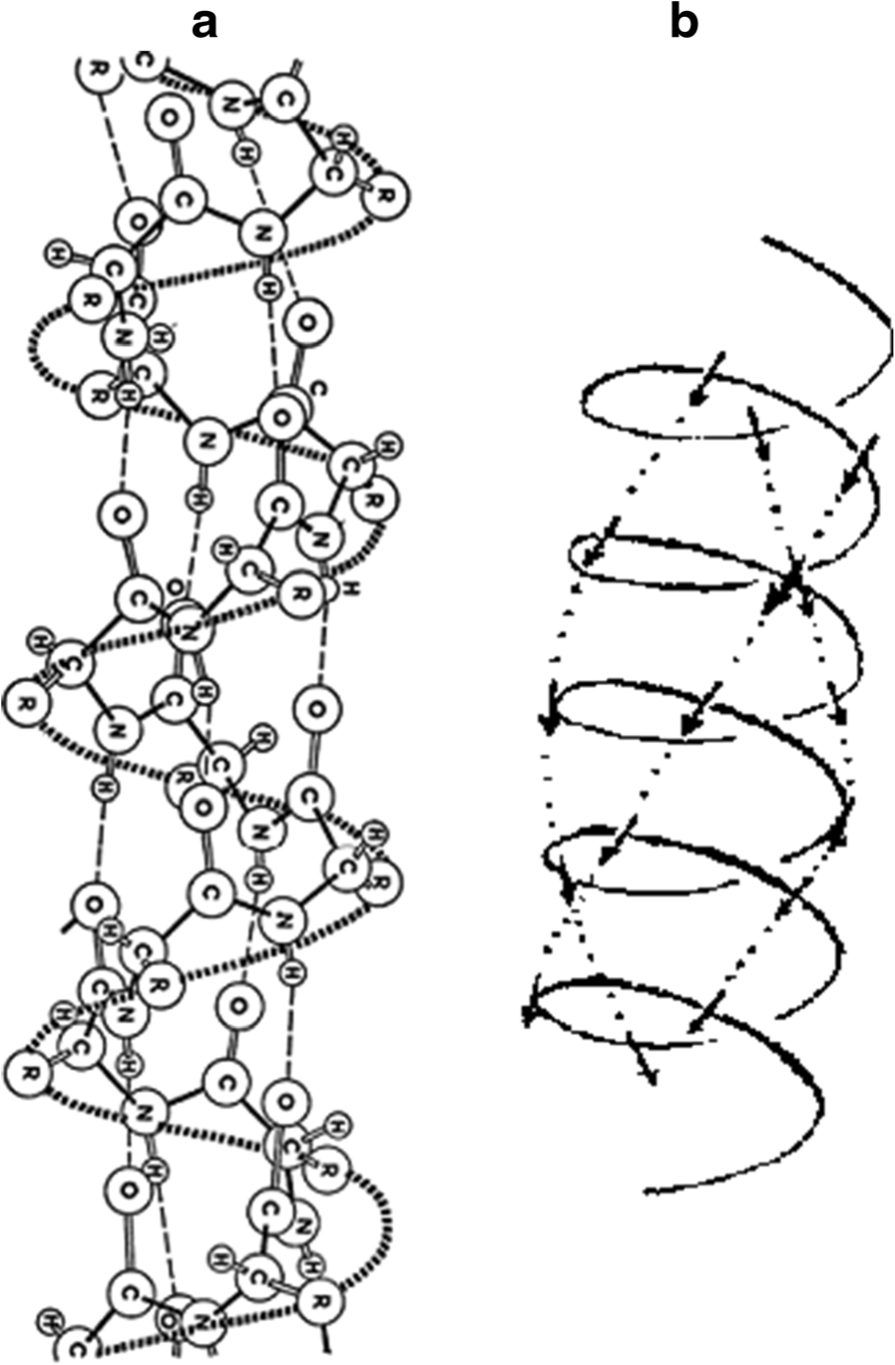
**The real (a) **[1][2]** and schematic (b) **[3]** images of an alpha-helix.**

Figure 1 shows the alpha-helical fragment of a protein molecule. Similar regions in proteins are widespread enough *in vivo*. The degree of helicity in different proteins varies from 12% to 96%. As can be seen from Figure 1, the alpha-helical fragment of protein molecules is structurally a nanotube. The same is true for its physical properties. Therefore, to such regions of protein molecules in their excited states, it is natural to apply methods that are specific for nanotubes.

As a result of hydrolysis of ATP molecule, energy is realized in the range 0.2 to 0.4 eV^a^. It depends on the charge state of the ATP molecule, in which the composition of the environment influences mainly (pH, etc.). The energy of hydrolysis is absorbed by an alpha-helical region of the protein molecule. It takes place due to internal vibrational excitations of the peptide groups (HCNO) in the state amide I. Its energy is also varied within the limits of 0.2 to 0.4 eV. These excitations induce a significant increase of dipole moments of the peptide groups, which is equal to 3.7 *D*, on 0.29 *D*[4,5].

There exists another point of view. Excitation of amide I may have an electronic nature. It may correspond to transitions between energy bands with principal quantum numbers that are equal to 2. The physical nature of excitation is inessential for further calculations, but further it will be shown that their nature may be determined experimentally.

## Methods

### Amide I excitation in the simplest model of alpha-helical region of protein

Foremost, we need to determine the model of description of the spatial structure of the alpha-helix. Since it is considered as a molecular crystal, the nearest neighbor approximation is used, which is typical for such crystals. However, as seen from Figure 1b, the nearest neighbors for some peptide group with number *n* are not only group *n* ± 1 but also group *n* ± 3.

**Figure 2:**
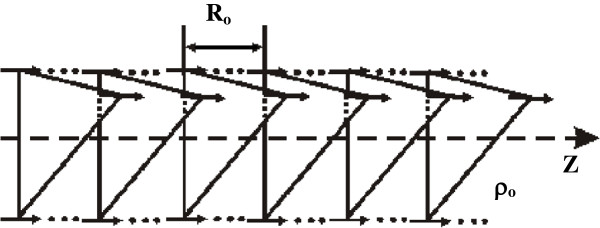
**Simplest model of alpha-helix as a one-dimensional molecular crystal with three molecules per unit cell.** Arrows are showing a separate peptide group. They symbolize the dipole moments.

The simplest model of the spatial structure of the alpha-helix is shown in Figure 2. Such simplified model differs from a real molecule only by symmetry. In the model considered, the molecule is independent from each other: translational and axial symmetries. The real molecule has translational-helical symmetry. Preliminary investigations have already shown that the qualitative picture in terms of types of excitation does not change. Changes will only be quantitative. They will lead only to some displacement of the absorbing states by energy and, may be, to some mixing of states. In this, simplest, model, all turns of the helix closed on itself, although Figure 1 shows that this is not quite so. Each turn of the helix is open for the nearest neighbor. It was previously shown [6] that taking into account open individual cells leads only to quantitative changes. The qualitative picture remains unchanged.

Within the framework of the considered model, every three peptide groups that belong to one turn of the helix grouped into one complex unit cell. We will number these unit cells by indices *n*, *m*, etc. The number of such cells is three times less than the number of peptide groups, i.e., *N*_0_/3. Peptide groups within a single cell will be enumerated by indices *α*, *β*, etc. that may take values 0, 1, 2. The general functional for the alpha-helix in this model has the form [7]

$$\begin{array}{l} E\left(\left\{R\right\},\left\{A\right\}\right)=\frac{1}{2}\sum_{n\;\alpha }\sum_{m\;\beta }\{w\left(R_{n\;\alpha }-R_{m\;\beta }\right)+\{D\left(R_{n\;\alpha }-R_{m\;\beta }\right){\left|A_{\alpha \;n}\right|}^{2}\\ \;+M_{n\;\alpha ,m\;\beta }A_{\;\beta \;m}^{*}A_{\;\alpha \;n}\}\}/. \end{array}$$

*w*(**R**_*nα*_ − **R**_*mβ*_) in this functional is the basic energy of interaction between peptide groups *nα* and *mβ*. It is independent on the presence of excitation and exists always. *D*(**R**_*nα*_ − **R**_*mβ*_)|*A*_*αn*_|^2^ is an additional energy to the *w*(**R**_*nα*_ − **R**_*mβ*_) energy of interaction related only to excitation but considerably smaller. Factor *A*_*αn*_ is the wave function that describes the excited state of the examined alpha-helical region of the protein molecule. It determines the spatial-temporal distribution of excitation in this region. The energy *D*(**R**_*nα*_ − **R**_*mβ*_)|*A*_*αn*_|^2^ leads to the breaking of the equilibrium of the alpha-helix and stimulates its conformational response to excitement. Energy $M_{\mathrm{n\alpha},\mathrm{m\beta}}A_{\beta \;m}^{*}A_{\mathrm{\alpha n}}$ is also an additional energy of interaction. However, it is much less than *D*(**R**_*nα*_ − **R**_*mβ*_)|*A*_*αn*_|^2^ but important because it provides the propagation and transfer of excitation along the alpha-helix.

As shown in Figure 2, the nearest neighbors for some peptide group *nα* will only be the peptide groups *m* = *n* ± 1, *β* = *α* and *m* = *n*, *β* = *α* ± 1. Taking into account that in the considered model all energy terms depend on the distances between amino acid residues only, the following formulae in the nearest neighbor approximation may be obtained: *R*_*nα*_ ≡ |**R**_*n* + 1,*α*_ − **R**_*n*,*α*_|, *ρ*_*nα*_ ≡ |**R**_*n*,*α* + 1_ − **R**_*n*,*α*_|.

Let us take into account that the response of the lattice (Figure 2) on excitation inside of the unit cell is small enough. Thus, it may be neglected in comparison with a similar response between unit cells. In this sense, the equality *ρ*_*nα*_ = *ρ*_0_ is always supposed fulfilled. Factor *R*_*nα*_ is the only value that takes into account the response of the alpha-helix on excitation. Thus, we will denote its equilibrium value as *R*_0_. Values *ρ*_0_ and *R*_0_ are shown in Figure 2. Taking into account the normalization condition

(1) $$\sum_{n\;\alpha }{\left|A_{\;n\;\alpha }\right|}^{2}=1,$$

the last functional takes the form

(2) $$\begin{array}{c} E\left(\left\{R\right\},\left\{A\right\}\right)=\sum_{\mathrm{n\alpha}}w_{⊥}+D_{⊥}+\sum_{\mathrm{n\alpha}}\left\{w\left(R_{\;n\;\alpha }\right)+D\left(R_{\;n\;\alpha }\right){\left|A_{\;\alpha \;n}\right|}^{2}+\right)\\ \left(+\frac{1}{2}M_{\left|\right|}\left(A_{\;\alpha ,n+1}^{*}+A_{\;\alpha ,n-1}^{*}\right)A_{\;\alpha \;n}+\frac{1}{2}M_{⊥}\left(A_{\;\alpha +1,n}^{*}+A_{\;\alpha -1,n}^{*}\right)A_{\;\alpha \;n}\right\}\;. \end{array}$$

Here, *w*_⊥_ ≡ *w*(*ρ*_0_), *D*_⊥_ ≡ *D*(*ρ*_0_), *M*_⊥_ = *M*(*ρ*_0_), and *M*_||_ = *M*(*R*_0_). Obviously, |*M*_⊥_| ≠ |*M*_||_|. If resonance interaction has no electronic nature, inequality will be realized: |*M*_⊥_| < |*M*_||_|. If excitation has an electronic nature, inequality will be reversed: |*M*_⊥_| > |*M*_||_|. This difference may be detected experimentally, and the answer of the question about the physical nature of excitation may be obtained.

New equilibrium values of distances, which actually coincide with the step of alpha-helices, are determined using the general condition of minimization: $\frac{\partial E\left(\left\{R\right\},\left\{A\right\}\right)}{\partial R_{\;n\;\alpha }}=0$. When interactions between peptide groups are modeled as purely dipole, the step of the alpha-helix always decreases and is given by

(3) $$R_{\;n\;\alpha }=R_{0}-\frac{\left|D^{/}\left(R_{0}\right)\right|}{\left|w^{//}\left(R_{0}\right)\right|}{\left|A_{\;n\;\alpha }\right|}^{2}.$$

Next, we must substitute (3) in (2), take into account the condition $\frac{\left|D^{/}\left(R_{0}\right)\right|}{\left|w^{//}\left(R_{0}\right)\right|}{\left|A_{\;n\;\alpha }\right|}^{2}<<R_{0}$, designate *w*(*R*_0_) ≡ *w*_||_, *D*(*R*_0_) ≡ *D*_||_, $\frac{{\left|D^{/}\left(R_{0}\right)\right|}^{2}}{\left|w^{//}\left(R_{0}\right)\right|}\equiv G$, and introduce convenient re-designation: *M*_||_ = −|*M*_||_| ≡ −2*Λ*, *M*_⊥_ = |*M*_⊥_| ≡ 2*Π*, which take into account the true signs. Then for the functional (2), finally, the following formula will be obtained:

(4) $$\begin{array}{l} E\left(\left\{A\right\}\right)=E_{\mathrm{осн}}-\sum_{\mathrm{n\alpha}}\{\Lambda \left(A_{\alpha ,n+1}^{*}+A_{\alpha ,n-1}^{*}\right)A_{\mathrm{\alpha n}}+\frac{1}{2}G{\left|A_{\mathrm{\alpha n}}\right|}^{4}\\ \;-\Pi \left(A_{\alpha +1,n}^{*}+A_{\alpha -1,n}^{*}\right)A_{\mathrm{\alpha n}}\}. \end{array}$$

In Equation 4, *E*_осн_ = (*w*_⊥_ + *w*_||_)*N*_0_ + *D*_⊥_ + *D*_||_, and the following is taken into account:

$$\sum_{n\;\alpha }\left(w_{⊥}+w_{\left|\right|}\right)=\left(w_{⊥}+w_{\left|\right|}\right)N_{0}.$$

*N*_0_ is the number of amino acid residues in the alpha-helical region of the protein molecule, which is under consideration.

Further, for implementation of the conditional minimization of energy (4) in relation to wave functions *A*_*αn*_, it is necessary to create a conditional functional: $E^{\mathrm{ум}}\left(\left\{A\right\}\right)=E\left(\left\{A\right\}\right)+\epsilon \left(1-\sum_{\mathrm{n\alpha}}{\left|A_{\mathrm{\alpha n}}\right|}^{2}\right)$. From a mathematical point of view, parameter *ϵ* is an indefinite Lagrange multiplier, and physically, it is the eigenvalue of the considered system. The minimization procedure $\frac{\partial E^{\mathrm{ум}}\left(\left\{A\right\}\right)}{\partial A_{\;\alpha \;n}^{*}}=0$ produces the equation *Λ*(*A*_*α*,*n* + 1_ + *A*_*α*,*n* − 1_) + *G*|*A*_*αn*_|^2^*A*_*αn*_ − *Π*(*A*_*α* + 1,*n*_ + *A*_*α* − 1,*n*_) + *ϵA*_*αn*_ = 0. After dividing this equation by *Λ* and introducing the notations,

(5) $$g\equiv \frac{G}{\Lambda }\;;\;\lambda \equiv \frac{\Pi }{\Lambda }\;;\;\chi \equiv \frac{\epsilon }{\Lambda },$$

it is possible to reduce it to a dimensionless form:

(6) $$A_{\;\alpha ,n+1}+A_{\;\alpha ,n-1}+g{\left|A_{\;\alpha n}\right|}^{2}\;A_{\;\alpha n}-\lambda A_{\;\alpha +1,n}-\lambda A_{\;\alpha -1,n}+\chi A_{\;\alpha n}=0.$$

The function *A*_*αn*_ is complex. Therefore, the common solution of the system (6) has the form *A*_*αn*_ = *a*_*αn*_ · exp(*iγ*_*αn*_). Amplitude *a*_*αn*_ and phase *γ*_*αn*_ are real functions of the variables *α* and *n*. We confine ourselves to the Hamiltonian-Lagrangian approximation in phase [8]. Due to the stationarity of the solved problem, this approximation has the simplest form: *γ*_*αn*_ ≡ *kn*. If the alpha-helical part of the molecule is long enough,^b^ a Born-Karman condition gives $k=\frac{2\pi }{N_{c}}j$. Here, $N_{c}\equiv \frac{N_{0}}{3}$ is the number of turns in the considered alpha-helical region of the protein molecule. It plays the role of the dimensionless length of the helical region of the protein in units of an alpha-helix step. Parameter *j* has the values $j\approx 0,\;\pm 1,\;\pm 2,\ldots ,\pm \frac{N_{c}}{2}$. Then

(7) $$A_{\;\alpha n}=a_{\;\alpha n}\;e^{\mathrm{ikn}},$$

and Equation 6 takes the form

$$a_{\;\alpha ,n+1}e^{\mathrm{ik}}+a_{\;\alpha ,n-1}e^{-\mathrm{ik}}+ga_{\;\alpha \;n}^{3}-\lambda a_{\;\alpha +1,n}-\lambda a_{\;\alpha -1,n}+\chi a_{\;\alpha \;n}=0.$$

Separating real and imaginary parts, we have the following formulae:

(8) $$\cos\left(k\right)\cdot \left(a_{\;\alpha ,n+1}+a_{\;\alpha ,n-1}\right)+ga_{\;\alpha \;n}^{3}-\lambda a_{\;\alpha +1,n}-\lambda a_{\;\alpha -1,n}+\chi a_{\;\alpha \;n}=0;$$

(9) $$\sin\left(k\right)\cdot \left(a_{\;\alpha ,n+1}-a_{\;\alpha ,n-1}\right)=0.$$

The solution of this system is usually determined after transition to continuous approximation. But we will analyze systems (8) and (9) without using the continuous approximation, because we are interested in very short alpha-helical regions (10 to 30 turns).

There is only condition *a*_*α*,*n* + 1_ − *a*_*α*,*n* − 1_ = 0 (if not to restrict solutions by using the condition *k* = 0), which does not depend on any symmetry of the alpha-helix: whether it is the symmetry of the model or the symmetry of the real molecule. Viewing of other conditions can appear useful on account of the real structure of the alpha-helical region. In the simplest case, it may be reduced to the equation *a*_*αn*_ = *P*_*α*_. The system (8) now degenerates in the system of three nonlinear equations:

(10) $$\begin{array}{c} xP_{0}-P_{1}-P_{2}+yP_{0}^{3}=0;\\ -P_{0}+xP_{1}-P_{2}+yP_{1}^{3}=0;\\ -P_{0}-P_{1}+xP_{2}+yP_{2}^{3}=0;\\ P_{0}^{2}+P_{1}^{2}+P_{2}^{2}=\frac{1}{N_{c}}, \end{array}$$

where the following designations are introduced:

(11) $$\frac{\chi +2\cos\left(k\right)}{\lambda }\equiv x;\;\frac{g}{\lambda }\equiv y.$$

The last, fourth, equation arose out from normalization condition (1). The coefficients *P*_*α*_ (*α* = 0, 1, 2) determine the excitement of each peptide chain as a whole.

The system (10) consists of four nonlinear equations for determining the values *P*_0_, *P*_1_, and *P*_2_ and the eigenvalue *x*. By adding and subtracting the first two equations and some transformation of the third equation, the system (10) can be reduced to the form

(12) $$\begin{array}{c} \left(P_{0}-P_{1}\right)\left[x+1+y\left(P_{0}^{2}+P_{1}^{2}+P_{0}P_{1}\right)\right]=0;\\ \left(P_{0}+P_{1}\right)\left[x-1+y\left(P_{0}^{2}+P_{1}^{2}-P_{0}P_{1}\right)\right]=2P_{2};\\ \left(P_{0}+P_{1}\right)=\left(x+yP_{2}^{2}\right)P_{2};\\ P_{0}^{2}+P_{1}^{2}+P_{2}^{2}=\frac{1}{N_{c}}. \end{array}$$

This transformation does not affect the solutions of the system.

For the solution, the condition *P*_0_ + *P*_1_ = 0 should be used. This condition together with the condition *P*_2_ = 0 turns into an identity the second and third equations. After some simple transformations, we obtain the *antisymmetric* excitations:

$$\begin{array}{l} P_{0}^{\left(a\right)}=\frac{1}{\sqrt{2N_{c}}};\;P_{1}^{\left(a\right)}=-\frac{1}{\sqrt{2N_{c}}};\\ P_{2}^{\left(a\right)}=0;\;x_{a}=-1-\frac{y}{2N_{c}}. \end{array}$$

Using Equations 4, 5, and 11, it is possible to find the energy:

(13) $$E_{a}\left(k\right)=E_{\mathrm{осн}}+\epsilon_{a}\left(k\right)=E_{\mathrm{осн}}-\Pi -\frac{G}{2N_{c}}-2\Lambda \cos\left(k\right).$$

Next, we use the condition *P*_0_ − *P*_1_ = 0, which turns into an identity the first equation in (12). After some analysis, we can find two types of excitation:

● *Symmetrical*

$$P_{0}^{\left(c\right)}=P_{1}^{\left(c\right)}=P_{2}^{\left(c\right)}=\frac{1}{\sqrt{3N_{c}}};\;x_{c}=2-\frac{y}{3N_{c}}.$$

● For these excitations, in analogy to the antisymmetric, it is possible to obtain the energy:

(14) $$E_{c}\left(k\right)=E_{\mathrm{осн}}+2\Pi -\frac{G}{3N_{c}}-2\Lambda \cos\left(k\right).$$

● *Asymmetrical*

$$\begin{array}{l} P_{0}^{\left(н\right)}=P_{1}^{\left(н\right)}=-\frac{1}{\sqrt{6N_{c}}};\\ P_{\;2}^{\left(н\right)}=\sqrt{\frac{2}{3N_{с}}}\;;\;x_{н}=-1-\frac{2y}{3N_{c}}. \end{array}$$

● For these excitations, it is also possible to get energy:

(15) $$E_{н}\left(k\right)=E_{\mathrm{осн}}-\Pi -\frac{2G}{3N_{c}}-2\Lambda \cos\left(k\right).$$

The energies *E*_*a*_(*k*), *E*_*c*_(*k*), and *E*_*н*_(*k*) contain parameters *Λ* = |*M*_||_|/2 and *Π* = |*M*_⊥_|/2. As it was noted between Equations 2 and 3, the relation between these parameters makes the determination of the physical nature of excitation possible: whether they are electronic or intramolecular. Because one of them (*Λ*) determines the width of the excited energy bands, and the other (*Π*) their positions, this is the basis for the experimental analysis of the nature of excitations.

There are a few possibilities else for searching for solutions of the system (12). Preliminary analysis shows that the obtained excitations are peculiar in a more or less degree for both symmetries: whether it is the symmetry of the model or the symmetry of the real molecule. The other solutions of the system (12) need to be analyzed only in the conditions of the maximum account of the real structure of an alpha-helix. But the general analysis of this system shows that the solutions of a new quality are not present: all of them belong to the asymmetrical type. However, attention should be paid to the equation *a*_*α*,*n* + 1_ − *a*_*α*,*n* − 1_ = 0, which has led to the requirement *a*_*αn*_ = *P*_*α*_. This condition is strong enough and essentially limits the solution: it is a constant in variable *n*, i.e., does not have the spatial distribution along an alpha-helix.

## Results and discussion

### The analysis of the energetics of the protein excitation

From definitions (13), (14), and (15), it ensues that received excitations are located in accordance with the inequality *E*_*c*_(*k*) > *E*_*a*_(*k*) > *E*_*н*_(*k*). Thus, $E_{c}\left(k\right)-E_{a}\left(k\right)=3\Pi +\frac{G}{6N_{c}}$ and $E_{a}\left(k\right)-E_{н}\left(k\right)=\frac{G}{6N_{c}}$. It can be seen that for the alpha-helical region of finite length, when the number of turns *N*_*c*_ ≠ *∞*, the lowest energy is the energy of asymmetric excitation *E*_*н*_. Also, it is visible that energy *E*_*c*_ is always strongly separated from energies *E*_*a*_ and *E*_*н*_. Even when the number of turns *N*_*c*_ ⇒ *∞* and the energies *E*_*a*_ and *E*_*н*_ practically coincide, the energy *E*_*c*_ is separated from *E*_*a*_ and *E*_*н*_ on a value 3*Π* = 3|*M*_⊥_|/2. Amide I excitations manifested experimentally are probably *E*_*c*_ energy.

It is possible to make the supposition that each of the examined energies executes some, expressly certain, function. For example, the main function of symmetric excitations can be activation of muscle proteins. At the same time, they can activate both membrane and enzymatic proteins that are quite often actually observed in the activation of myosin [9-11].

Antisymmetric excitation energy is not enough to excite the muscle protein because it lies below the symmetric energy. Activation of membrane proteins can be their main function. At the same time, these excitations are able to activate enzymatic proteins that are also actually observed often enough during activation of membranes [11-13].

And, lastly, asymmetrical excitations have only one function - to activate exceptionally enzymatic activity in those cases, when membrane and muscular activities are not needed. That is only for intracellular processes.

### Conformational response to the excitation of the alpha-helical region of the protein molecule

For the analysis of conformational response of the alpha-helix on the considered excitations, it is necessary to appeal again to new equilibrium values of the step of the alpha-helix. From definition (3), it is possible to find *R*_*nα*_ = *R*_0_ · (1 − *β*|*A*_*αn*_|^2^), where designation is entered: $\beta \equiv \frac{\left|D^{/}\left(R_{0}\right)\right|}{R_{0}\cdot \left|W^{//}\left(R_{0}\right)\right|}$. If we consistently apply the model of dipole interaction between the peptide groups, then $\beta \sim \frac{\mathrm{\Delta d}}{d}$, where, as mentioned above, Δ*d* ~ 0.29 *D* and *d* ~ 3.7 *D*. Therefore, in this dipole model [14], *β* ~ 10^−1^. Taking into account the definitions of coefficients *A*_*αn*_, given in (7), it is possible to get following:

1. It is possible to obtain the following formula for symmetric excitations: $R_{\mathrm{n\alpha}}^{\left(c\right)}=R_{0}\cdot \left(1-\frac{\beta }{3N_{c}}\right)$. That is, all three chains are reduced equally and evenly in the space. Then the length of every peptide chain can be appraised, so

$$L_{\alpha }^{\left(c\right)}=\sum_{n=1}^{N_{c}}R_{\mathrm{n\alpha}}^{\left(c\right)}\equiv N_{c}R_{0}-\frac{1}{3}\beta R_{0}\equiv L_{0}-\frac{\beta R_{0}}{3}.$$

This change is small and, at first glance, has no practical significance. But it will be so only in the classical model of the alpha-helix (Figure 2). If we consider, for example, that the peptide chains of myosin themselves form superhelices, then the effect of contraction increases. This is done by changing all characteristics of an alpha-helix: the step of the helix, its radius, and the effective number of peptide groups on the turn of the helix. Also, additional self-torsion takes place. The strengthening of the effect of contraction is determined by the mutual torsion of long alpha-helical regions of light faction of myosin and their torsion on actin filaments.

2. For antisymmetric excitations, it is possible to obtain $R_{n0}^{\left(а\right)}=R_{n1}^{\left(а\right)}=R_{0}\cdot \left(1-\frac{\beta }{2N_{c}}\right)$, $R_{n2}^{\left(а\right)}=R_{0}$. Respective lengths are as follows:

$$L_{0}^{\left(a\right)}=L_{\;1}^{\left(a\right)}=L_{0}-\frac{\beta R_{0}}{2};\;L_{2}^{\left(a\right)}=L_{0}\equiv R_{0}N_{c}.$$

**Figure 3:**
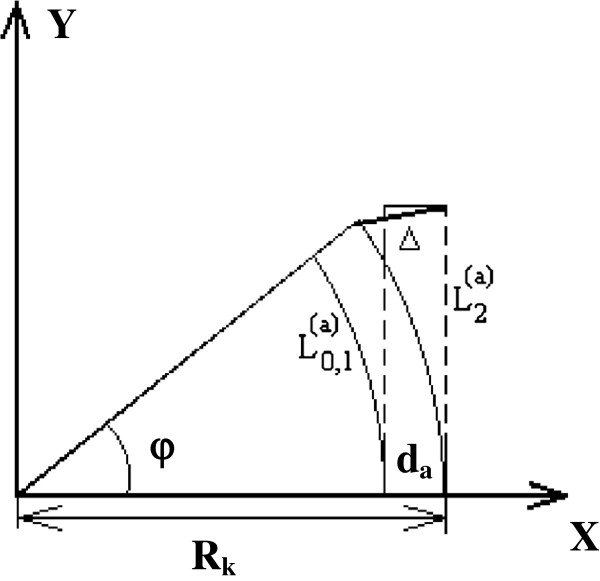
Explanation to estimation of displacement Δ of free (here upper) end of alpha-helix for antisymmetric excitations.

In this type of excitation, one of the peptide chains does not change (here, it is a chain with the number 2), and two others are reduced up to the value $\frac{\beta R_{0}}{2}$. Such asymmetry is enough for the alpha-helix to take a form of the segment of torus instead of cylinder (Figure 3). Application of the simple geometric considerations gives for the radius of curvature *R*_*k*_ and angle *φ*:

$$R_{k}\equiv \frac{2}{\beta }\cdot \frac{d_{\alpha }}{R_{0}}\cdot L_{0}\equiv \frac{2}{\beta }\cdot d_{\alpha }\cdot N_{c};\;\varphi \equiv \frac{\beta }{2}\cdot \frac{R_{0}}{d_{\alpha }},$$

and for displacement Δ, it is possible to get such estimation:

(16) $$\Delta \sim \frac{\beta }{2\sqrt{3}}\cdot \frac{R_{0}}{d_{\alpha }}\cdot L_{0}=\frac{\beta }{2\sqrt{3}}\cdot \frac{R_{0}^{2}}{d_{\alpha }}\cdot N_{c}.$$

Taking into account the numerical values *β* ~ 10^−1^, *R*_0_ = 5.4 Å, and *d*_*α*_ = 4.56 Å in (16) gives $\Delta \sim \frac{N_{c}}{5}\;\left(Å\right)$. For the typical number of turns in many enzymes and membrane squirrel (*N*_*c*_ > 10), displacement will have an order Δ > 2 Å. This is consistent with the observed values [11].

3. For asymmetrical excitation, the following values are implemented: $R_{n0}^{\left(н\right)}=R_{n1}^{\left(н\right)}=R_{0}\cdot \left(1-\frac{\beta }{6N_{c}}\right)$, $R_{n2}^{\left(н\right)}=R_{0}\cdot \left(1-\frac{2\beta }{3N_{c}}\right)$. The corresponding lengths of peptide chains equal

$$L_{0}^{\left(н\right)}=L_{1}^{\left(н\right)}=L_{0}-\frac{\beta R_{0}}{6},\;L_{2}^{\left(н\right)}=L_{0}-\frac{2\beta R_{0}}{3}.$$

The nature of the distribution of deformation along the peptide chain for this type of excitation is similar to that of the antisymmetric excitation. The only difference is that the chain, which in the previous case has not changed at all, now has shortening stronger than the other two. It is possible to estimate displacement for this case too:

$$\Delta^{\left(н\right)}=\Delta \;\sqrt{1-\frac{\beta }{6N_{c}}+\frac{1}{3N_{c}^{2}}{\left(\frac{d_{\alpha }}{R_{0}}\right)}^{2}}.$$

Here, Δ is the displacement for antisymmetric excitations, which is determined by Equation 16. Unlike displacement Δ, displacement Δ^(*н*)^ ‘directed’ to the opposite side. Executing numerical estimates, it is possible to set that Δ^(*н*)^ > Δ, if the number of turns in the alpha-helix *N*_*c*_ ≤ 14, but at *N*_*c*_ > 14, we will have Δ^(*н*)^ < Δ accordingly.

Consequently, asymmetrical excitations demonstrate two very interesting features. First, it has the lowest energy and at diminishment of the number of turns *N*_*c*_, it falls down yet more. Second, a conformational response for this type of excitation is the biggest for *N*_*c*_ ≤ 14. This is typical for enzymatic proteins only.

## Conclusions

The general methods [7,15-17] of description of the excited states of the condensed environments were applied to the alpha-helix region of a protein molecule. The alpha-helix is considered as a nanotube, and excitations of the environment are described as quasiparticles. It is shown that three different types of excitation exist, and each of them is probably used by three different types of protein. The symmetrical type of excitation is used for muscle proteins, the antisymmetric type of excitation is used for membrane proteins, and the asymmetric type of excitation is used for enzymatic proteins. It is possible that some excitations of asymmetrical type exist, which are also used by enzymes. The estimations were done for displacements of the free end of the alpha-helix. The obtained displacements are in agreement with experimental data. Therefore, the obtained results can be the basis of the interpretation of the functional properties of proteins characterizing their activity related to their conformational changes [11].

## Endnotes

^a^Off-system unit of energy: 1 eV = 1.602 × 10^−19^ J.

^b^For example, in the myosin protein, the helical region has about 200 turns or up to 700 amino acids.

## Competing interests

The authors declare that they have no competing interests.

## Authors’ contributions

AD Suprun. He formulated a scientific problem, analyzed the results and take part in the discussing and formulating the conclusions. LV Shmeleva. She made mathematical calculations, take part in the discussing of the results and conclusions. Both authors read and approved the final manuscript.
